# O07 Sarcoidosis: it's bone deep

**DOI:** 10.1093/rap/rkaa053.006

**Published:** 2020-11-03

**Authors:** Basil Noureldin, David Parr, Timothy Woo, Nicola Gullick

**Affiliations:** r1 Rhuematology: University Hospitals Coventry & Warwickshire, Coventry, United Kingdom; r2 Respiratory Medicine: University Hospitals Coventry & Warwickshire, Coventry, United Kingdom; r3 Radiology: University Hospitals Coventry & Warwickshire, Coventry, United Kingdom

## Abstract

**Case report - Introduction:**

This is a report of a Caucasian patient with sarcoidosis presenting as groin pain.

**Case report - Case description:**

A 58-year-old Caucasian female from South Africa self-referred for physiotherapy following sudden onset right groin pain. Pelvic Xray was normal, but MRI pelvis showed lytic lesions in the acetabulum and proximal femur. The presence of night sweats suggested haematological malignancy leading to haematology referral. PET CT showed widespread metabolically active soft tissue lesions, lymph nodes and skeletal foci. Bone marrow trephine biopsy showed well-formed granulomata. An extensive infection screen was negative. A cervical lymph node biopsy and CT guided biopsy of pelvic and femoral lesions confirmed non-necrotising granulomas suggestive of sarcoidosis. Lung function tests were normal. Treatment with 30mg prednisolone for one month was poorly tolerated and steroids were rapidly weaned. She was unable to tolerate even low dose prednisolone. Severe pelvic pain and night sweats continue despite methotrexate, hydroxychloroquine and IV zoledronate (given for osteoporosis).

**Case report - Discussion:**

This patient presented with groin pain, fatigue and night sweats, no demonstrable extra-osseous organ abnormality and normal plain radiography. Our diagnostic strategy was to first exclude malignancy and infection, particularly TB, but the unusual presentation created significant diagnostic uncertainty. Treatment is challenging due to intrusive side effects of steroids and hydroxychloroquine, and neutropenia induced by methotrexate.

Osseous involvement is rare and has been reported in 1-15% of sarcoidosis patients. It is usually accompanied by skin lesions. There were no features on history or examination to suggest sarcoidosis and the diagnosis was based on bone biopsies.

In a case series of 20 patients with osseous sarcoidosis, bone lesions were found on imaging during the initial presentation in 60% of cases. Ten patients were symptomatic, and all had multiple joint involvement. Axial involvement, primarily pelvis and lumbar spine was seen in 90% of cases. Lesions were detected on plain radiographs in 2 cases and were identified on MRI in 13 cases, PET-CT in 9 cases, CT in 4 cases and technetium-99m bone scintigraphy in 1 case. 55% of the cases required no treatment and 45% were treated most commonly with prednisolone, methotrexate, or hydroxychloroquine. Two cases required treatment with tumour necrosis factor inhibitors for refractory disease.

**Case report - Key learning points:**

Osseous sarcoidosis can mimic many conditions including multiple myeloma and lymphoma, due to presentation with lytic lesions. Even pulmonary presentations can be atypical with predominant ground glass opacity or cavitating consolidation and parenchymal masses. Histopathological diagnosis is of critical importance, particularly in patients with atypical presentations.

Assessment of organ involvement and disease extent is important in monitoring of treatment response. Plain radiographs tend to be normal in these cases and advanced imaging, including MRI, PET-CT, and CT, is often required.

Symptomatic osseous disease may respond to steroids and conventional immunosuppression; a minority of refractory cases require TNF inhibition.

